# Case Report: Pulmonary Metastases From Epithelioid Sarcoma in Extremity Favourably Responding to Immunotherapy With Camrelizumab

**DOI:** 10.3389/fonc.2021.728437

**Published:** 2021-10-06

**Authors:** Tao-Jun Gong, Fan Tang, Chuan-Xi Zheng, Jie Wang, Yi-Tian Wang, Ya-Han Zhang, Yi Luo, Yong Zhou, Li Min, Chong-Qi Tu

**Affiliations:** ^1^ Department of Orthopeadics, West China Hospital, Sichuan University, Chengdu, China; ^2^ State Key Laboratory of Biotherapy and Cancer Center, West China Hospital, Sichuan University, Chengdu, China; ^3^ Department of Pathology, West China Hospital, Sichuan University, Chengdu, China

**Keywords:** epithelioid sarcoma, pulmonary metastases, tumour immune microenvironment, immune checkpoint inhibitor, PD-L1

## Abstract

Epithelioid sarcoma (ES) is a rare soft tissue sarcoma (STS), with limited therapies available for metastatic disease. Here, we describe a case of a 30-year-old male with ES of the left knee and underwent surgery and radiation therapy for the primary disease. After 2 years, he had local recurrence and underwent extensive resection surgery; however, adjuvant chemotherapies were delayed due to recurrent wound infection. Nine months after the second surgery, progressive disease was confirmed after detection of metastases to the lungs and inguinal lymph nodes. Amputation was performed for the local recurrence, followed by inguinal lymph nodes dissection. Pazopanib was transiently administered but discontinued as a result of wound dehiscence. The tumour specimens were detected with unexpected high level of PD-L1 expression and tumoural infiltrating lymphocytes. Subsequently, he received camrelizumab 2.0 mg/kg every 21 days for 18 cycles with rapid remission of the pulmonary metastases. This promising response to camrelizumab indicates that immunotherapies may be an alternative choice for patients with metastatic ES in lung based on analysing the tumour immune microenvironment.

## Introduction

Epithelioid sarcoma (ES) is an extremely rare subtype of malignant mesenchymal tumour that accounts for less than 1% of all soft tissue sarcomas (STSs) (1). This type of sarcoma usually occurs in young to middle-aged adults with an average age of 20–40 years (2). Histologically, over 90% of epithelioid sarcomas lose the expression of integrase interactor 1 (INI-1), resulting in oncogenic dependence on enhancer of zeste homolog 2 (EZH2) (3, 4). Surgical resection is regarded as the only beneficial treatment for patients with local ES, with an overall survival rate of over 60% (5). However, the high recurrence rate after surgical resection and the poorer survival of regional disease when compared with local tumours warrants more effective therapies than surgery. Distant metastases of ES commonly occurred in lungs, and particularly in lymph nodes which were more frequent than other sarcomas (6). Furthermore, the reported high metastatic rate of 20%–50% in patients with ES indicates a dismal overall survival, with none of the patients with metastatic disease having a survival of over 5 years (1, 6, 7).

Currently, there are limited therapies with high efficacy for advanced or metastatic ES. Conventional chemotherapy with anthracycline or gemcitabine only achieved a median progression-free survival (PFS) of 4–6 months in late-stage ES (8). The role of targeted therapy using drugs with small molecules in ES is of increasing interest. Pazopanib was the first tyrosine kinase inhibitor approved for ES in a salvage setting. However, the results are not satisfactory, with only 3 months of PFS in 18 patients with advanced ES treated with pazopanib (8). Recently, based on the typical loss of expression of INI-1/SMARCB1, the Food and Drug Administration approved tazemetostat, an EZH2 inhibitor, for patients with advanced ES (9). However, the objective responses to date have mostly been partial, with 9/62 (15%) patients having an objective response (10). Even though, due to its therapeutic potential, a phase 1b/3 trial of tazemetostat plus doxorubicin in the front-line setting is underway (NCT04204941). However, tazemetostat is currently not available in China.

Immunotherapy has anti-tumour efficacy in certain types of late-stage solid tumours (11). The response rate of STS to immune checkpoint inhibitors (ICI) have met with mixed results, with an overall response rate of 15% to anti-PD-1 therapy (12). However, clinical trials have suggested that among sarcomas, alveolar soft part sarcoma, undifferentiated pleomorphic sarcoma (UPS), and dedifferentiated liposarcoma respond relatively well to ICI (13–15). A recent study revealed that the distinctive tumour immune microenvironments (TIME) of each type of sarcoma are mechanisms underlying the different responses to anti-PD-1 therapy (16). In addition, pulmonary metastases of sarcoma were detected have a better tumoural microenvironment regarding the response to immunotherapy than the primary tumour (17). However, due to the rarity of ES, limited studies have analysed the tumour immune microenvironment as well as the response to immune checkpoint inhibition. Camrelizumab (SHR-1210), a highly-affinity, humanized, IgG4-κ PD-1 monoclonal antibody against PD-1 originally yielded by China, has a clinically therapeutic effect on some solid tumours including osteosarcoma, Hodgkin’s lymphoma, advanced hepatocellular carcinoma, non-small cell lung cancer, and esophageal cancer (18, 19). Nevertheless, no studies have reported results regarding camrelizumab in ES patients. Here, we report the case of a 30-year-old man with refractory metastatic ES with a high level of PD-L1 expression. The patient achieved a rapid remission after the administration of the PD-1 inhibitor camrelizumab, after unsuccessful surgery and pazopanib therapy. The TIME of this case was analysed.

## Case Presentation

**Figure 1:**
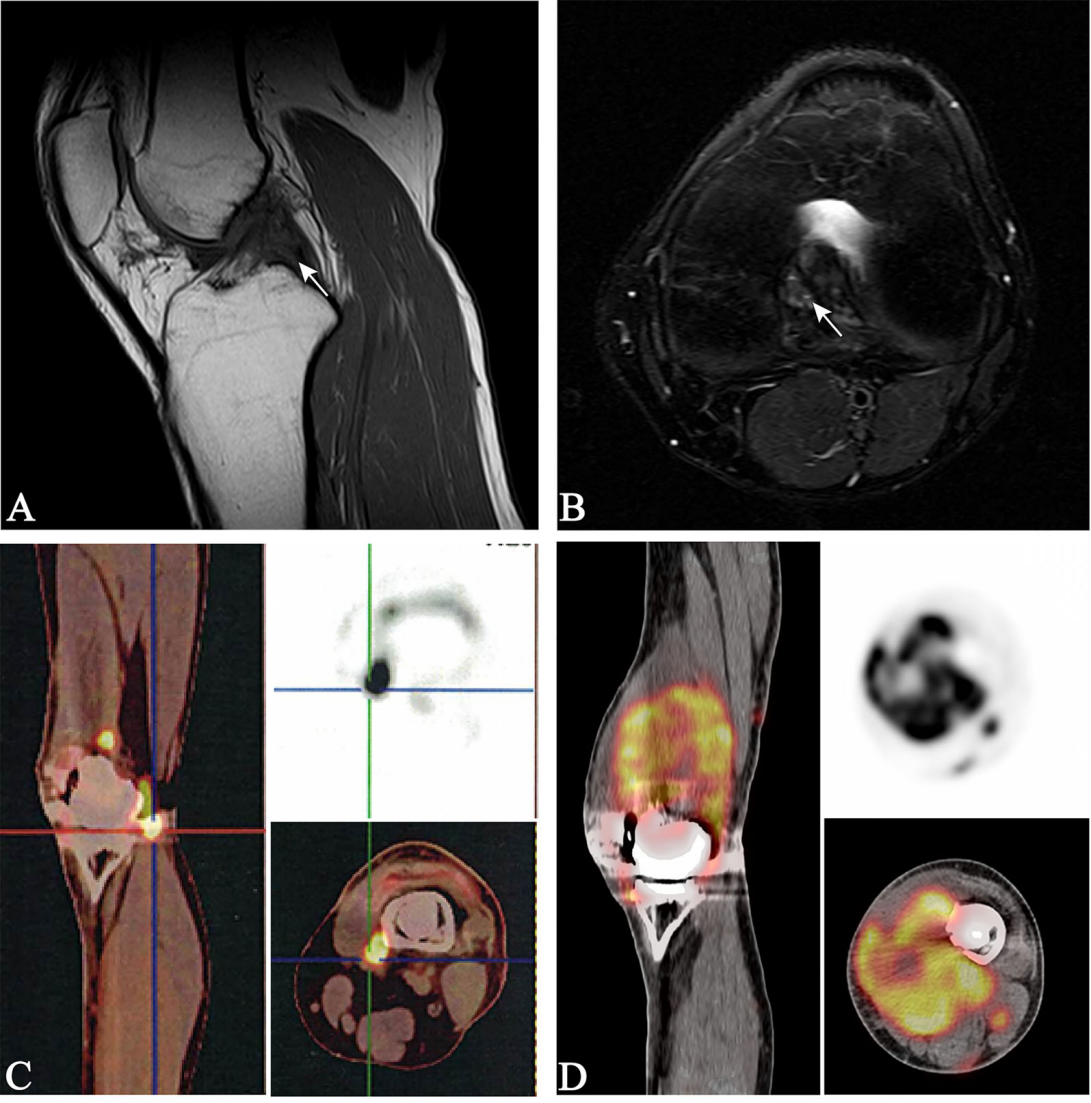
Magnetic resonance imaging of the left knee **(A, B)**, white arrows indicate a mass measuring 2.8 × 3.8 cm violating the cruciate ligaments; positron emission tomography-computed tomography (PET-CT) **(C)**, the fluorodeoxyglucose-avid tumour is seen within the left knee, indicating tumour recurrence; PET-CT **(D)**, the fluorodeoxyglucose-avid tumour is presented on maximum intensity projection image as increased uptake around the left knee, indicating a second tumour recurrence.

**Figure 2:**
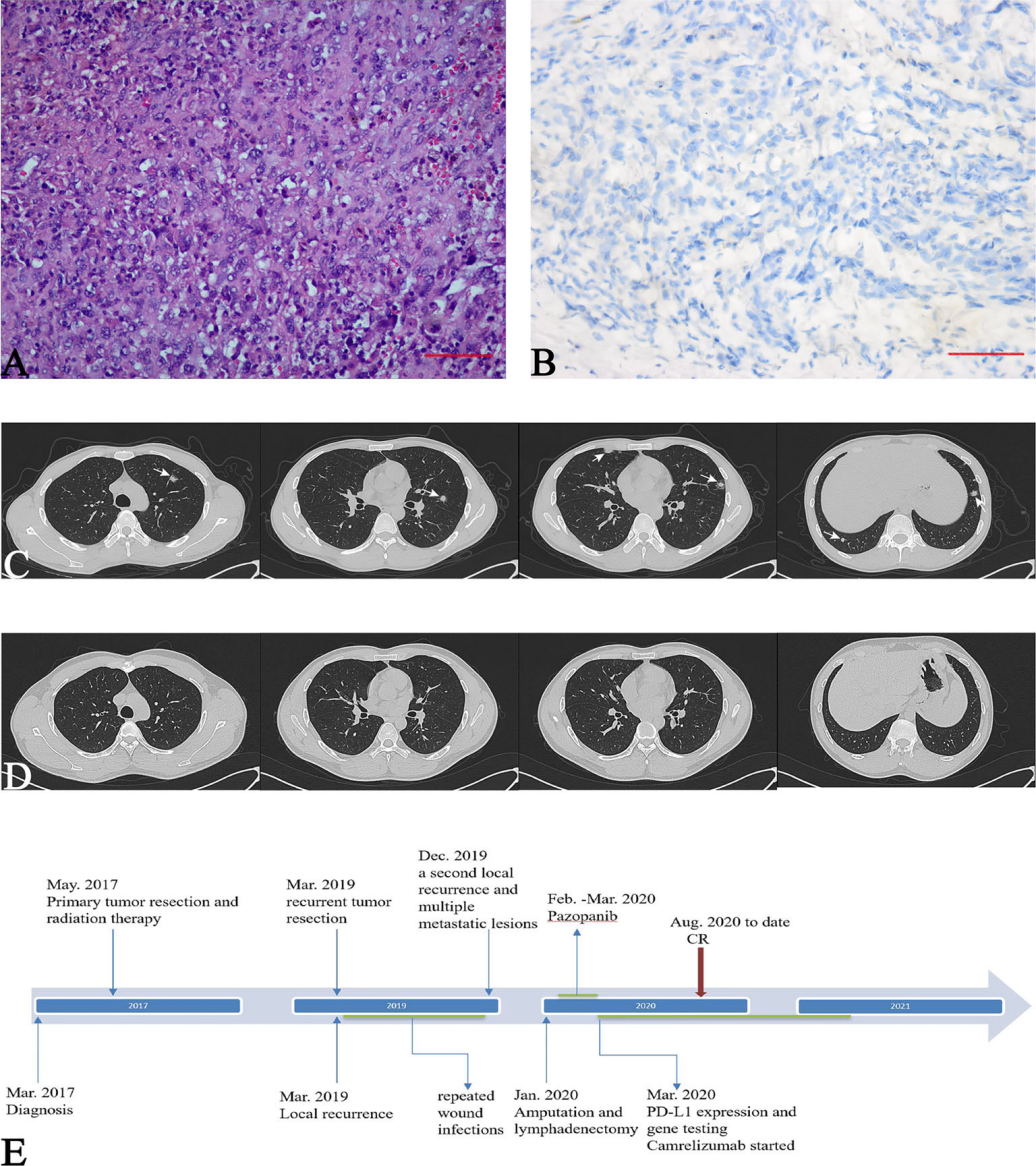
Pathological features of the local lesions. **(A)** Sheets of epithelioid and spindle-shaped large atypical cells and moderate nuclear pleomorphism (Haematoxylin and eosin stain, 200×); CT of the chest, **(B)** Immunohistochemical loss of integrase interactor 1 (INI-1) (400×); **(C)** white arrows indicate multiple pulmonary metastases **(D)** five months after camrelizumab therapy, pulmonary metastases completely disappeared; **(E)** Timeline of treatments from diagnosis to date.

A 30-year-old man who presented with left knee pain was referred to our hospital in March 2017. Magnetic resonance imaging (MRI) showed a mass measuring 3.8 cm × 2.8 cm that violated the cruciate ligaments (**Figures 1A, B**). Biopsy under arthroscopy was performed, and pathology revealed the diagnosis of ES, with no expression of INI-1 (**Figures 2A, B**). In May 2017, the patient underwent wide resection of the lesion, followed by endo-prosthetic reconstruction. After surgery, the patient received field radiation therapy at a dose of 60 Gy. The pain of knee was relieved after surgery and radiotherapy. However, 2 years later, a palpable mass was detected in the left popliteal fossa in March 2019. Positron emission tomography-computed tomography (PET-CT) indicated tumour recurrence with a maximum 8.4 SUV activity in areas of prior radiation therapy, whereas, no distant metastasis was detected (**Figure 1C**). The patient underwent a tumour-wide resection with negative margins. Postoperative pathology confirmed disease recurrence. After the second procedure, the patient experienced recurrent wound infection, and he underwent repeated debridement. Therefore, adjuvant chemotherapy was delayed.

In December 2019, the patient presented to our hospital in a severe condition with complaints of cough and haemoptysis. Physical examination revealed a cachectic patient with an Eastern Cooperative Oncology Group (ECOG) performance status of 3. PET-CT demonstrated a second local recurrence with a maximum 7.6 SUV activity, and multiple metastatic lesions in the lungs and left inguinal lymph nodes (**Figures 1D**, **2C**), maximum SUV activity in 4.8.

**Figure 3:**
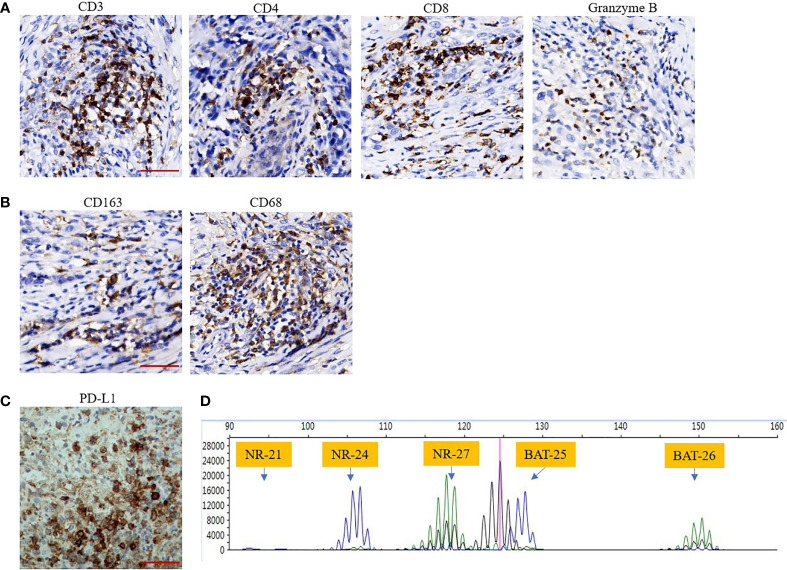
**(A, B)** Tumour immune microenvironment (TIME) analysis indicates that TILs including CD3^+^, CD4^+^, CD8^+^ cells and TAMs including CD68^+^, CD163^+^ cells are enriched in TIME. Granzyme B also was detected foci positive in TIME (immunohistochemical staining, Scar bar 50 μM, 400×); **(C)** High expression of PD-L1 as assessed by 30% (immunohistochemical staining, Scar bar 50 μM, 400×); **(D)**, PCR-based microsatellite instability testing using five microsatellite markers (BAT25, BAT26, NR-21, NR-24, NR-27), revealed microsatellite status of stable.

Amputation and lymphadenectomy were performed after evaluation by multidisciplinary team based on the following facts, first, the recurrent tumour presented with high tumour burden in local area, and the tumour mass had ruptured and diabrosis formed over the tumour surface, which resulted in endoprosthesis reconstruction failure. At this condition, amputation would benefit for tumour control and tumour burden reduction; Second, metastatic sarcomas received limb tumour resection are proposed to have better overall survival and improvement of quality of life in short life expectancy (20, 21). Following salvage therapy, oral pazopanib (800 mg daily) was administered in February 2020; however, after 30 days of therapy, wound dehiscence occurred. Therefore, pazopanib was discontinued. TIME analysis and polymerase chain reaction (PCR)-based microsatellite instability (MSI) testing were conducted. Immunohistochemical (IHC) staining of the resected recurrent tumour with 22C3 pharmDx assay (Agilent Technologies, Santa Clara, California, USA) revealed over 30% of PD-L1 positivity (**Figure 3C**). Moreover, CD3^+^ and CD8^+^ lymphocytes were enriched in TIME (**Figure 3A**). In addition, Granzyme B, which is secreted by cytotoxic T cells or NK cells, also detected foci positive in TIME (**Figure 3A**). Macrophages related markers including CD163 and CD68 were also detected (**Figure 3B**). MSI testing revealed the microsatellite status of stable (MSS) (**Figure 3D**).

Considering the possible response to ICI based on the TIME, after comprehensive assessment by a multidisciplinary team, a single agent, camrelizumab (Jiangsu Hengrui Medicine Co., Ltd. China) was administrated at 2 mg/kg every 21 days since March 2020. Subsequent chest CT scans demonstrated a durable response of the pulmonary lesions. In August 2020, that 5 months after commencing camrelizumab, pulmonary metastases demonstrated a complete response (CR) (**Figure 2D**). In March 2021, after completing 18 cycles of camrelizumab, the patient showed a durable response. Regarding the toxicities of camrelizumab therapy, the patient developed grade 1 reactive cutaneous capillary endothelial proliferation, which was well-tolerated. The patient was asymptomatic and had a normal physical examination with an ECOG performance status of 0 at the time of writing (**Figure 2E**).

## Methods

### Tumour Immune Microenvironment (TIME) Analysis

TIME was analysed to explore the potential therapeutic target and possible mechanisms of the favourable response to anti-PD-1 therapy in our patient. The evaluated parameters of TIME include tumour-infiltrating lymphocytes (TILs), expression of PD-L1, and the stability of microsatellite markers. The antibodies used for immunohistochemical (IHC) staining are: CD3 (CST, 85061S, USA), CD4 (Abcam, ab101530, USA), CD8 (CST, 85336S, USA), CD163 (Abcam, ab182422, USA), CD68 (Abcam, ab955, USA), Granzyme B (CST, 46890S, USA), and PD-L1 (Abcam, sp142, USA). Polymerase chain reaction (PCR)-based microsatellite instability (MSI) testing using five microsatellite markers (BAT25, BAT26, NR-21, NR-24, and NR-27).

## Discussion

Clinical management of patients with ES, especially late-stage or advanced disease, is challenging because of the limited therapies available. Immunotherapy using ICI has shown increasing therapeutic potential for solid tumours, including metastatic soft tissue sarcomas such as alveolar soft part sarcoma and UPS (14, 22). ES treated with ICI (some concurrent with or following pazopanib) were reported sporadically before, and clinic benefits were observed in small group of patients (14, 15, 23). Thus, an in-depth analysis of the factors influencing the response rate and screening for biomarkers that indicate good efficacy after anti-PD-1 therapy is essential (24). Furthermore, due to the high heterogeneity of sarcomas, analysis of the TIME of individual cases, especially late-stage disease or extremely rare subtype such as ES, may benefit these groups of patients.

Although tumour mutation burden (TMB) was not measured in our case, data of TMB is expected to be low to moderate in ES (10, 15). Microsatellite stability was confirmed using PCR, which is in agreement with the previously reported case of a metastatic ES having a MSS (15). This patient with end-stage ES had a rapid and complete response to the combined anti-CTLA-4 and anti-PD-1 checkpoint inhibitor therapy (15). Apparently, a single biomarker cannot precisely predict the response to immune checkpoint inhibition. In addition, the evaluation of TMB does not account for the host immune response or tumour microenvironment (24). Thus, composite biomarkers assessing other factors in conjunction with TMB or MSI may have a stronger positive predictive value (25).

The late-stage ES in our study have a relatively 30% of PD-L1 expression. In soft tissue sarcomas, positive PD-L1 expression in tumour specimens was observed in 12% to 58% cases, demonstrating a relatively low PD-L1 expression rate and the high heterogeneity of STS (26–28). The degree of PD-L1 positivity was correlated with a more advanced disease, higher risk of metastasis, and poor clinical outcome (28). Based on this clinical significance, high PD-L1 expression predicts a doubled response rate compared to tumours showing no staining (29, 30). For example, UPS, which has a favourable response to anti-PD-1 therapy, has higher levels of PD-L1 compared to other subtypes of sarcomas (31). In the phase 2 SARC028 trial, pembrolizumab was used as a single agent in relapsed sarcomas and had an ORR of 18%. The response was most noticeable in UPS, with a 40% ORR including one CR and three PRs of the 10 evaluable patients (12).

The late-stage tumour in our case presented with a high infiltration of TILs, which is an independent prognostic factor in microsatellite stable solid tumours (32). This was supported by a recent clinical evidence that regardless of the tumour type, the presence of TILs was strongly associated with the clinical outcome in patients who received immunotherapies instead of MSI status (33). In addition, PD-L1 expression was confirmed to be associated with the infiltration of CD4^+^ and/or CD8^+^ T cells, indicating that 98% of PD-L1^+^ tumours were associated with TILs, with 28% of PD-L1 negative tumours (34). More importantly, TILs and PD-L1 positivity in tumours would predict a robust response to anti-PD-1 therapy.

In our case, the primary tumour typically lost the expression of INI-1. Despite low TMB and microsatellite stability, tumours with INI-1 deficiency significantly express PD-L1, as demonstrated by 47% (14/30) of the INI-1-negative tumours that expressed PD-L1. In addition, 60% (18/30) of INI-1-negative tumours are CD8^+^ (35). Mechanisms underlying the increased tumour immunogenicity of INI-1 deficient tumours were that 89% of cases had SMARCB1 alterations, leading to the inactivation of SMARCB1 and the subsequent deficiency of the SWI/SNF chromatin remodel complex. SMARCB1-dependent interferon-signalling activation was proposed to mediate the increase in INI-1-deficient tumour immunogenicity (36). Overall, the clinical tumour tissue staining indicates that INI-1-deficient tumours may respond well to anti-PD-1 therapy, similar to the ES reported in our study. In a study by Forrest et al., three patients with INI-1-negative tumours had disease control after the administration of ICI (35).

It is supposed that the metastatic disease harbours a higher expression of PD-L1 than the primary disease. In 10 patients with osteosarcoma, after multi-region sequencing between primary tumours and the corresponding pulmonary metastases, metastatic tumours were proven to have higher neoantigens, increased expression of PD-L1, and elevated presence of TILs than the corresponding primary tumours (17). The increase in PD-L1 expression in metastatic or recurrent tumours enhances the potential benefits of ICI in patients with metastatic disease, as the case in our study (37). Finally, whether prior transient administration of pazopanib facilitated the rapid remission of pulmonary metastases in our case, remains unknown. This would be a subject worthy of further exploration, as multiple agents initiate, remodel, and promote the immune response that provides therapeutic benefits in patients with solid tumours (38).

## Conclusion

We described a patient with pulmonary metastases of ES, in which the tumoural immune microenvironment indicates a possibly response to anti-PD-1 immunotherapy. After the administration of camrelizumab, the pulmonary metastases of this patient were rapidly remised. This case in our study demonstrates a complete response of ES to a PD-1 inhibitor and ICI may benefit selected patients with pulmonary metastases from this rare type of sarcoma in extremity.

## Data Availability Statement

The original contributions presented in the study are included in the article/supplementary material. Further inquiries can be directed to the corresponding authors.

## Ethics Statement

The studies involving human participants were reviewed and approved by the West China Hospital’s ethics committee, Sichuan University. The patients/participants provided their written informed consent to participate in this study.

## Author Contributions

T-JG and FT contributed equally to this work and are co-first authors. T-JG analysed and interpreted the patient case, T-JG and FT wrote and edited the manuscript. C-XZ, JW, Y-TW, YZ, and YL reviewed the manuscript. LM and C-QT supervised this case report and reviewed the manuscript. All authors contributed to the article and approved the submitted version.

## Funding

This work was supported, in part, by the National Natural Science Foundation of China (No. 82002847), the National Key Research and Development Program of China (No. 2017YFB0702604), the Science and Technology Research Program of Sichuan Province (No. 2017SZ0095).

## Conflict of Interest

The authors declare that the research was conducted in the absence of any commercial or financial relationships that could be construed as a potential conflict of interest.

## Publisher’s Note

All claims expressed in this article are solely those of the authors and do not necessarily represent those of their affiliated organizations, or those of the publisher, the editors and the reviewers. Any product that may be evaluated in this article, or claim that may be made by its manufacturer, is not guaranteed or endorsed by the publisher.

## Abbreviations

ES, epithelioid sarcoma; STS, soft tissue sarcoma; PD-1, programmed death 1; PD-L1, programmed death-ligand 1; CTLA-4, cytotoxic T-lymphocyte-associated protein 4; UPS, undifferentiated pleomorphic sarcoma; TIME, tumour immune microenvironment; CR, complete response; PET-CT, positron emission tomography-computed tomography; ECOG, Eastern Cooperative Oncology Group; TILs, tumour-infiltrating lymphocytes; TAMs, tumour-associated macrophages; IHC, immunohistochemical; TMB, tumour mutation burden; MSI, microsatellite instability; MSS, microsatellite status of stable.
